# Monocular Markerless Motion Capture Enables Quantitative Assessment of Upper Extremity Reachable Workspace

**DOI:** 10.3390/s26113421

**Published:** 2026-05-28

**Authors:** Seth Donahue, J.D. Peiffer, R. Tyler Richardson, Yishan Zhong, Shaun Q. Y. Tan, Benoit L. Marteau, Stephanie A. Russo, May D. Wang, R. James Cotton, Ross Chafetz

**Affiliations:** 1 Shriners Children’s Lexington, Lexington, KY 40508, USA; 2 Department of Physical Therapy, University of Kentucky, Lexington, KY 40536, USA; 3 Shirley Ryan AbilityLab, Center for Bionic Medicine, Chicago, IL 60611, USA; johnpeiffer2026@u.northwestern.edu (J.D.P.); rcotton@sralab.org (R.J.C.); 4 Department of Physical Medicine and Rehabilitation, Northwestern University, Evanston, IL 60208, USA; 5 Department of Kinesiology, School of Behavioral Sciences and Education, Pennsylvania State University at Harrisburg, Middletown, PA 17057, USA; rtr12@psu.edu; 6 School of Electrical and Computer Engineering, Georgia Institute of Technology, Atlanta, GA 30332, USA; yzhong307@gatech.edu (Y.Z.); stan99@gatech.edu (S.Q.Y.T.); benoitmarteau@gatech.edu (B.L.M.); 7 Nationwide Children’s Hospital, Columbus, OH 43205, USA; 8 Wallace H. Coulter Department of Biomedical Engineering, Georgia Institute of Technology and Emory University, Atlanta, GA 30322, USA; maywang@gatech.edu; 9 Shriners Hospitals for Children, Philadelphia, PA 19140, USA; rchafetz@shrinenet.org

**Keywords:** reachable workspace, Markerless Motion Capture, monocular camera, clinical feasibility, artificial intelligence

## Abstract

This study validates a clinically accessible approach for quantifying the Upper Extremity Reachable Workspace (UERW) using monocular AI-driven Markerless Motion Capture (MMC). Objective validation of such techniques for clinically oriented tasks is essential to support their adoption in clinical motion analysis. Nine adults without impairments performed the standardized UERW task, reaching targets distributed across a virtual sphere centered on the torso and displayed via VR headset. Movements were simultaneously captured with a marker-based system and eight FLIR cameras; monocular analysis was applied to two videos representing frontal and offset camera configurations. Agreement was assessed by comparing the percentage workspacereached across six of eight workspace octants between the systems. The frontal camera demonstrated strong agreement with the marker-based reference (mean bias: 0.61±0.12% reachspace per octant), whereas the offset view underestimated workspace reached −5.66±0.45%. Depth-related errors in the frontal configuration were confined to posterior octants, whereas the offset view introduced inaccuracies in both contralateral and posterior octants. These findings support the feasibility of a frontal monocular camera for UERW assessment, particularly for anterior workspace evaluation. While posterior accuracy remains limited by depth estimation and anatomical occlusion errors, the overall results demonstrate clinical potential for practical, monocular-camera assessments.

## 1. Introduction

Quantitative tracking of human movement is central to clinical assessment, enabling identification of biomechanical phenotypes that complement subjective evaluations and patient-reported outcomes (PROs) [1,2]. Traditionally, this tracking has been performed using marker-based motion capture, which is considered the gold standard for biomechanical analysis. Marker-based systems track anatomical landmarks using retro-reflective markers and have been widely used to assess upper extremity (UE) function, with high spatial accuracy of ≤2 mm [2,3].

Over the past two decades, UE motion assessment has evolved from subjective scoring to objective quantification using marker-based systems [4,5,6]. However, many analyses have focused on a limited set of motions or static poses [7,8]. In contrast, the Upper Extremity Reachable Workspace (UERW) provides a holistic appraisal of global UE mobility by quantifying the regions in space that can be reached by the hand [3,9]. The UERW task involves patients reaching toward virtual targets displayed around them, providing a quantitative, biofeedback-driven measure of their reachable workspace [2]. Assessment of the UERW task is calculated by the number of targets reached compared to the number of targets available in the octant to reach and is expressed as a percentage. The previous literature has established UERW as a valid, objective and clinically relevant measure to differentiate between patient populations and healthy controls, and to track rehabilitation progress over time [2,10,11,12]. While highly accurate and clinically valuable, the measurement of UERW with marker-based systems comes with several practical challenges. This includes the need for specialized laboratory space, multiple cameras, careful calibration, and technical expertise for both setup and operation. Variability in marker placement between clinicians and skin motion artifacts may affect accuracy. In addition, patients often require significant preparation time, and data processing can be labor-intensive. Together, these factors make it challenging to integrate motion capture into everyday clinical workflows, limiting its accessibility for many patients and clinicians.

As an alternative to marker-based motion capture, MMC has emerged as a promising alternative. Initial work in this space included simple video-based methods for objective movement analysis using manual annotation of patient joints and body segments, often performed frame by frame by an engineer or clinical team member [13,14,15]. However, these manual approaches are time-intensive and require both clinical and technical expertise. Advances in artificial intelligence (AI), computer vision, and human pose estimation now offer a pathway to overcome these limitations; multi-camera systems, such as Theia3D, OpenCap, and open-source biomechanics frameworks, have demonstrated high accuracy, with errors in the sagittal plane $\leq 5^{\circ }$, in angle estimations across diverse movement tasks [16,17,18,19,20,21]. Despite the accuracy of multi-camera MMC for UE assessment, clinical implementation remains hampered by the complexity of the synchronized hardware [17,22,23,24,25]. Given the logistical challenges of multi-camera systems, monocular MMC offers a simpler, more scalable approach for point-of-care biomechanical analysis [26,27]. Recent advancements have demonstrated that monocular reconstructions can achieve accuracy levels comparable to marker-based systems in controlled environments, even when accounting for inherent challenges such as occlusion and keypoint detection noise [28,29].

While frameworks utilizing computer vision keypoints have proliferated, many lack the underlying biomechanical constraints requisite for clinical decision making. Without these constraints, pose estimation often functions as a “black box” model, prioritizing 2D image fitting over the physical plausibility required for clinical trust [30,31,32]. Other portable alternatives, such as depth-sensing cameras, have demonstrated accuracy for upper extremity measurements but are limited by their need for specific lighting, specialized hardware, and a dedicated spatial footprint. Similarly, inertial measurement units (IMUs) offer portability but remain prone to sensor drift and magnetic interference in clinical settings.

Recently, monocular camera applications have successfully integrated anatomically accurate biomechanical models, such as MuJoCo, to overcome these limitations [27]. These emerging technologies hold the promise of delivering the capabilities of a biomechanics laboratory through a single mobile device. Currently, however, the technical validation of these systems has been largely confined to gait. Unlike the cyclical and relatively predictable nature of walking, upper extremity tasks involve higher degrees of freedom, significant self-occlusion, and rapid multi-planar transitions that challenge the limits of monocular pose and depth estimation [33,34].

To address this gap, the present study evaluates the technical validity of a monocular, biomechanically constrained MMC pipeline for the UERW task. This work represents a novel expansion of monocular MMC validation beyond linear locomotion into complex, volumetric clinical assessments. We evaluate two independent camera configurations—frontal and offset—against the clinical gold standard (marker-based motion capture). We hypothesize that the frontal configuration will demonstrate superior agreement across the six UERW octants, as the frontal perspective better preserves the primary planes of motion for the reaching task, minimizing the depth-perception discrepancies in the monocular camera view during dynamic reaching tasks.

## 2. Materials and Methods

### 2.1. Participants

A total of 9 adults without movement impairments (5 males, 4 females; height: 1.69 ± 0.09 m, mass: 70.56 ± 19.05 kg, right arm length: 0.57 ± 0.03 m) participated in this study. All participants were free of underlying conditions affecting upper extremity mobility and had no history of neurological injury within the past year. Only the UERW of the right arm was assessed, regardless of hand dominance.

### 2.2. Data Collection

**Figure 1:**
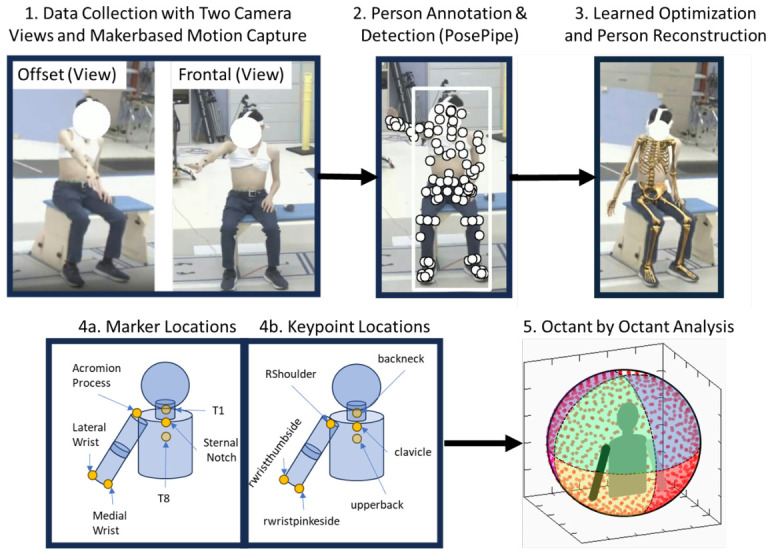
Methods overview. (**1**) Two monocular camera configurations (frontal and offset) were evaluated against marker-based motion capture. (**2**) Video processing included pose detection (MeTRAbs) and tracking (mmDeepSort). (**3**) Biomechanical reconstruction was performed using a MuJoCo-based optimization pipeline. (**4**) Correspondence between marker-based anatomical landmarks (**4a**) and keypoint locations (**4b**). (**5**) Reachable workspace was quantified as the percentage workspace reached within six anatomically defined octants (anterior/posterior, superior/inferior, ipsilateral/contralateral). Posterior contralateral octants were excluded from analysis.

For each participant, video- and marker-based data were collected simultaneously using a twelve-camera marker-based motion capture system (Vicon Industries, Hauppauge, NY, USA) and an eight-camera video recording system (Blackfly S Forward-Looking Infrared or FLIR, Teledyne, Thousand Oaks, CA, USA) through Vicon Nexus, sampled at 60 Hz. Data collection was conducted within a dedicated Motion Analysis Center, where ambient lighting and camera geometry were specifically optimized for high-fidelity kinematic tracking. Systems were hardware-synchronized through Vicon Nexus at 60 Hz to ensure temporal alignment for frame-by-frame agreement analysis. A gamified version of the UERW real-time feedback was presented using a virtual reality (VR) headset, the Meta Quest 3 (Meta, Menlo Park, CA, USA), with pass-through vision [35]. Participants viewed an array of virtual targets, placed at arm’s length as estimated by the VR system, with 800 targets superimposed over the pass-through VR view, and were instructed to reach for the targets with their right hand; for technical details, please see [35]. When the hand moved within 5 cm of a virtual target, the target disappeared. Participants were instructed to reach for all available targets until the full reachable volume was exhausted. To preserve the natural movement, participants were not constrained to specific predefined trajectories or reach patterns. Analysis was limited to the six octants defining the anterior and ipsilateral reachable workspace (Figure 1); the posterior contralateral octants were excluded from measurement in accordance with established UERW protocols [2,10].

The analysis presented in this work is a post hoc evaluation of two monocular views from the original eight FLIR video cameras analyzed independently. A frontal camera was positioned anterior and superior to the participant, while an offset view was positioned anterior, superior, and lateral, approximately 45° to the right of the frontal camera (Figure 1). We analyzed these two views independently using only camera intrinsics to emulate a monocular camera setup, following [27]. A standard wand-based calibration was performed for the laboratory space. Retro-reflective markers were affixed to the skin of the participants using adhesive Velcro coins; all markers were placed by the same trained researcher to ensure consistency. Markers were affixed to the radial and ulnar styloids of the right wrist, the acromion process of the right scapula, the T1 and T8 spinous processes, and the sternal notch (Figure 1). A static trial was subsequently recorded in Vicon Nexus to register the marker set and compute each participant’s maximum reach, but it was not needed for the monocular reconstructions.

### 2.3. Kinematic Processing

#### 2.3.1. Marker-Based Processing

The origin of the local torso coordinate system was defined as the midpoint between the sternal notch and T1, with the vertical vector V extending from T1 to T8. A placeholder vector in the anterior–posterior (AP) direction, ${\mathrm{AP}}_{\mathrm{temp}}$, was defined from T8 to the sternal notch. The medial–lateral (ML) vector, ML, was computed as the cross-product of ${\mathrm{AP}}_{\mathrm{temp}}$ and V: $\mathrm{ML}={\mathrm{AP}}_{\mathrm{temp}}\times V$. The final AP vector was computed as AP=V×ML, forming a right-handed, anatomical torso coordinate system. The end effector at the wrist was defined as the midpoint between the medial and lateral wrist markers. This differs from previous UERW work, which used a marker on the second digit as the end effector [2,10]. To ensure fair system comparison and more robust tracking in the monocular pipeline and minimize variance associated with finger keypoint estimations, the wrist midpoint was defined as the end effector for workspace estimation; while this yields more conservative volume measurements than second-digit models, it provides a more stable index of gross reaching kinematics in a monocular markerless framework.

The primary clinical outcome of the UERW is the % of workspace reached per octant. Targets were defined as the maximum distance of the wrist from the origin of the local torso coordinate system. Analysis was performed across six of the eight octants: superior anterior ipsilateral (Sup. Ant. Ipsil.), superior anterior contralateral (Sup. Ant. Contra.), inferior anterior contralateral (Inf. Ant. Contra.), inferior anterior ipsilateral (Inf. Ant. Ipsil.), inferior posterior ipsilateral (Inf. Post. Ipsil.), and superior posterior ipsilateral (Sup. Post. Ipsil.).

#### 2.3.2. Markerless Processing

Video data from both camera configurations were processed and 2D and 3D keypoints were extracted using MeTRAbs-ACAE, which outputs a superset of anatomical landmarks. From this set, we utilized the 87 MoVi keypoints, which provide robust coverage across anatomical landmarks relevant to biomechanical reconstruction [20,36,37]. This setup yields a purely monocular video-based system; that is, the biomechanical reconstructions from the monocular camera are based solely on 2D and 3D keypoint data extracted from a single video camera. Person detection and identity association were performed using mmdetDeepSort [38].

Biomechanical Reconstruction. Following the methods described by Peiffer et al. in the Portable Biomechanics Laboratory, we fit a whole-body biomechanical model in MuJoCo to the keypoints extracted from videos [27]. We express the forward kinematic mapping of the model as

(1) x=M(θ,β)

where $\theta \in R^{40}$ are joint angles, $\beta \in R^{8+87\times 3}$ contains eight body-scaling parameters and the keypoint offsets, and $x\in R^{87\times 3}$ and gives the resulting keypoint positions. MuJoCo’s GPU-accelerated engine enables parallelized forward-kinematic evaluations. The kinematic trajectory for each trial is represented as a learned implicit function $f_{\phi }$, implemented as a multi-layer perceptron with sinusoidal temporal encoding [39]:

(2) $$f_{\phi }:t\mapsto \theta \left(t\right)$$

Rotational outputs are passed through a tanh nonlinearity and linearly rescaled to biomechanical joint limits before being evaluated by the forward kinematic model in Equation (1). Each trial is associated with a dedicated implicit function, and we jointly optimize all trial-specific parameters ${\left\{\phi_{i}\right\}}_{i=0}^{N-1}$ together with the shared scaling and offset parameters β, following the bilevel-optimization approach of [40]. During each optimization step, 300 time samples per trial are evaluated to produce predicted joint angles $\hat{\theta }\left(t\right)$, followed by GPU-accelerated forward kinematic evaluations $\hat{x}=M\left(\hat{\theta }\left(t\right),\hat{\beta }\right)$. The 3D loss is defined as

(3) $$L_{3D}=\frac{1}{J}\sum_{j\in J}c\left(j\right)\;g\;\left({∥{\hat{x}}_{n}-x_{c}∥}_{2}\right),$$

where ${\hat{x}}_{n}$ are the 3D keypoints measured from the model at each optimization step, $x_{c}$ are the 3D keypoints extracted from the video, c(j) is the confidence score of each keypoint, and g(·) is a Huber loss (quadratic within 10 cm).

Using the calibrated camera intrinsics and projection model Π, the 2D loss compares projected model keypoints with detected 2D keypoints *u*:

(4) $$L_{2D}=\frac{1}{J}\sum_{j\in J}c\left(j\right)\;g\;\left({∥\Pi ({\hat{x}}_{n})-u∥}_{2}\right),$$

with a 5-pixel quadratic region for the Huber loss.

Although the original pipeline was designed for use with mobile devices with an inertial loss term, we have omitted this as our cameras in this work were static. Therefore, the full objective is

(5) $$L=\lambda_{1}L_{3D}+\lambda_{2}L_{2D},$$

with $\left(\lambda_{1},\lambda_{2}\right)=\left(1,10^{-1}\right)$. Training used Equinox, JAX, and Adam optimization with a learning-rate decay from $10^{-3}$ to $10^{-6}$ and weight decay of $10^{-5}$ [41,42]. Model training was completed using JAX 0.6.0 on a workstation with 64 GB RAM and an NVIDIA RTX 5000 GPU (16 GB VRAM) [20,27]. Post-processing was performed on a standard research workstation with a total inference and optimization time of approximately 1 h per participant with approximately 2 min videos. While this study prioritized technical validation over throughput, the current pipeline was not optimized for real-time clinical reporting.

All assessments of participant movement were performed post hoc. Coordinate systems derived from the MMC data were aligned with the marker-based coordinate systems, as the MoVi keypoint locations closely matched the anatomical positions of the marker-based system (Figure 1). The origin of the MMC coordinate system was defined as the midpoint between the “clavicle” and “backneck” keypoints, corresponding approximately to the sternal notch and T1 markers in the marker-based system. The vertical vector (V) extended from the “upper back” keypoint (approximately T8) to the “back of neck” keypoint (approximately T1). The placeholder AP vector, ${\mathrm{AP}}_{\mathrm{temp}}$, was defined from the “upper back” to the “clavicle”. The ML vector was calculated as $\mathrm{ML}={\mathrm{AP}}_{\mathrm{temp}}\times V$, and the final AP vector was AP=V×ML, forming a right-handed anatomical coordinate system. The calculation of maximum reach distance was identical to that used in the marker-based system.

### 2.4. Statistical Analysis

We assessed the performance of monocular MMC throughout the UE workspace using three measurements: (1) peak reach distance differences between marker-based and monocular MMC for each camera configuration; (2) the validity of the percentage workspace reached derived from monocular MMC; and (3) the agreement between the marker-based and monocular MMC systems in each octant at every time point.

First, we assessed differences in peak reach distance, measured as the distance from the origin of the torso coordinate system to the wrist in a single frame. This value determines the location of the virtual targets for post hoc analysis. We then computed the percentage workspace reached in each octant for the marker-based system and both MMC configurations.

Second, to assess statistical differences between systems and the six octants regarding the reachable workspace accessed, we used a two-way repeated-measures analysis of variance (ANOVA). Greenhouse–Geisser corrections were applied when sphericity was violated, and Bonferroni corrections were used for post hoc comparisons. The within-subjects factor “system” had three levels: marker-based, frontal, and offset. The within-subjects factor “octant” had six levels: superior anterior ipsilateral (Sup. Ant. Ipsil.), superior anterior contralateral (Sup. Ant. Contra.), superior posterior ipsilateral (Sup. Post. Ipsil.), inferior anterior ipsilateral (Inf. Ant. Ipsil.), inferior anterior contralateral (Inf. Ant. Contra.), and inferior posterior ipsilateral (Inf. Post. Ipsil.). The dependent variable was the percentage workspace reached in each octant. The alpha level was set at α=0.05, and all analyses were conducted in Python v3.12 using the Pingouin package (v0.5.5).

Finally, we assessed whether the marker-based and MMC systems agreed on which octant the end effector occupied at each time point. The rate of agreement was calculated as the percentage of time points where both systems assigned the wrist to the same octant:

$$\mathrm{Agreement}\;\mathrm{Rate}=\frac{\mathrm{Total}\;\mathrm{Agreements}}{\mathrm{Total}\;\mathrm{Time}\;\mathrm{Points}\;\mathrm{in}\;\mathrm{Octant}}\times 100\%.$$

When disagreement occurred, the direction of disagreement was identified, and a directional error rate was computed as follows:

$$\mathrm{Directional}\;\mathrm{Error}\;\mathrm{Rate}=\frac{\mathrm{Total}\;\mathrm{Disagreements}\;\mathrm{in}\;a\;\mathrm{Direction}}{\mathrm{Total}\;\mathrm{Time}\;\mathrm{Points}\;\mathrm{in}\;\mathrm{Octant}}\times 100\%.$$

For example, if the marker-based system measured the end effector in the Sup. Post. Ipsil. octant while the monocular MMC system placed it in the Sup. Ant. Ipsil. octant at the same time point, that frame would be recorded as a disagreement for the Sup. Post. Ipsil. octant. Consequently, the error direction would be identified as the AP direction for the calculation of the directional error rate.

## 3. Results

Differences in peak reach between the marker-based and MMC systems were minimal. Specifically, the differences in estimated reach distances were 0.04 ± 0.03 m for both the offset and frontal camera configurations.

**Figure 2:**
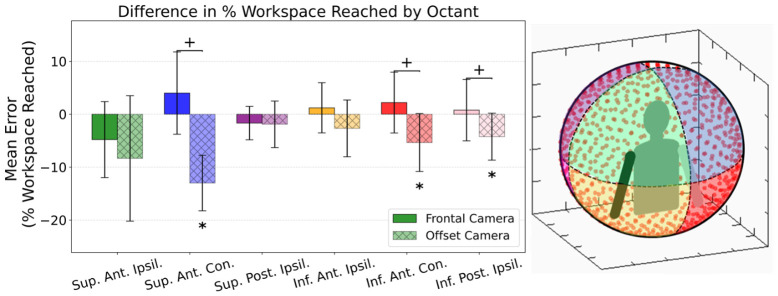
Differences in percentage workspace reached for each octant between the systems, where 0 indicates perfect agreement, positive values indicate overestimation, and negative values indicate underestimation of reachable workspace. An (*) indicates a statistically significant difference in the percentage workspace reached between either of the MMC camera configurations and the marker-based workspace in total percentage workspace reached (based on pairwise comparisons with Bonferroni correction, α=0.05). We further assessed the differences in percentage workspace reached between the two orientations and the marker-based system to identify specifically where the system disagreed. A (+) indicates a statistically significant difference between the frontal and offset camera configurations in % of workspace reached, highlighting where the two systems were not in alignment. A schematic of the six octants that the participants were accessing is shown on the right panel, and defined as relative to the torso-centered coordinate system. Corresponding colors and regions are mapped between the panel and the far right figure with the octants.

The total percentage workspace reached by each system is presented in (Table 1). The differences (MMC − marker-based) in the percentage workspace reached are presented in Figure 2. A two-way repeated-measures ANOVA on the percentage workspace reached in each of the six octants analyzed revealed significant main effects of the system $\left(F\left(2,16\right)=43.87,\;p<0.001,\;\eta_{G}^{2}=0.090\right)$ and octant $\left(F\left(5,40\right)=223.84,\;p<0.001,\;\eta_{G}^{2}=0.924\right)$, as well as a significant system × octant interaction $\left(F\left(10,80\right)=4.80,\;p=0.022,\;\eta_{G}^{2}=0.066\right)$. Bonferroni-corrected post hoc comparisons showed that the offset camera differed significantly in percentage workspace reached from both the frontal camera (t(8)=−6.91,p<0.001,g=−0.92) and the marker-based system (t(8)=−5.95,p=0.001,g=−0.68). In contrast, there was no significant difference between the frontal camera and marker-based systems overall (t(8)=2.16,p=0.189,g=0.24). Pairwise comparisons of the percentage of reachable workspace accessed revealed several significant differences across the camera configurations for specific octants (Table 2). Crucially, when comparing the frontal camera system against the marker-based system, no statistically significant differences in the percentage of reachable workspace were observed across any of the six measured octants (all p>0.303). The effect sizes for these frontal/marker comparisons were consistently small ($\eta_{p}^{2}$ ranging from 0.068 to 0.300).

In contrast, the offset camera system demonstrated a statistically significant difference compared to the marker-based system only in the superior anterior contralateral octant ($p<0.001,\;\eta_{p}^{2}=0.854$), indicating a large effect. Furthermore, significant differences were found between the two MMC systems (offset/frontal) in the inferior anterior contralateral ($p<0.001,\;\eta_{p}^{2}=0.892$) and superior anterior contralateral ($p<0.001,\;\eta_{p}^{2}=0.886$) octants, suggesting a substantial directional disparity between the two monocular configurations in the contralateral workspace.

Bland–Altman analysis revealed minimal differences in percentage workspace reached between the marker-based and frontal camera configurations (Table 3) and larger differences for the offset camera configuration. For the frontal camera, mean differences ranged from −4.44% to 4.56%. The Sup. Ant. Contra. octant demonstrated the largest positive bias (4.56%), whereas the Sup. Ant. Ipsil. octant showed the largest negative bias (−4.44%). The 95% confidence intervals (CIs) for individual octants with the frontal camera spanned from −18.55% to 19.29% (Table 3).

The offset camera configuration exhibited greater underestimation of reachable workspace compared to the marker-based system, with an overall mean difference across all octants of −5.59% (95% CI: −19.90% to 8.71%). Octant-specific mean differences ranged from −19.90% to −1.78%, with CIs spanning −31.34% to 15.34%. The Sup. Ant. Contra. octant showed the largest negative bias (−12.44%). There were no positive differences for the offset camera; on average, this configuration underestimated the percentage workspace reached, indicating fewer targets reached compared to the marker-based system (Table 3).

**Figure 3:**
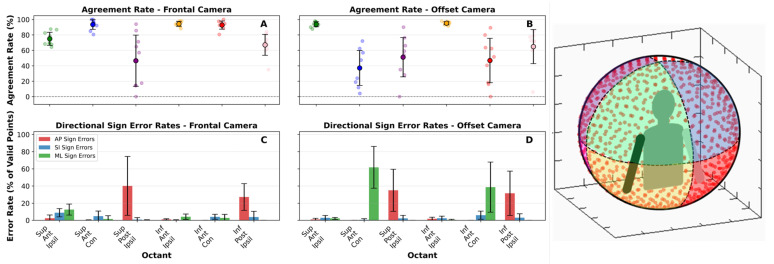
Agreement rate between the marker-based system and both MMC orientations (frontal and offset) across all octants. Panels (**A**,**B**) show the agreement rate between the two systems (% of frames where both systems assigned the same octant), with dot color matching their respective regions in the far right panel. Panels (**C**,**D**) show directional error rates when disagreement occurred, categorized by anatomical axes (anterior–posterior, superior–inferior, medial–lateral). Agreement of 100% indicates perfect classification consistency. Directional error rates represent the proportion of misclassified frames attributable to each anatomical direction. Lower agreement in posterior octants is associated with increased anterior–posterior depth ambiguity. A schematic of the six octants the participants were accessing is shown on the right panel, and defined as relative to the torso-centered coordinate system.

Agreement rates and directional error rates varied across octants and between the frontal and offset camera configurations (Figure 3). For the Sup. Ant. Ipsil. octant, the offset camera demonstrated higher agreement with the marker-based system (93.83±3.49%) compared to the frontal camera (74.97±8.99%), with generally lower error rates across the anterior/posterior, superior/inferior, and medial/lateral directions. In the Sup. Ant. Contra. octant, the frontal camera demonstrated superior agreement (93.48±6.67%) compared to the offset configuration (37.21±23.95%), which suffered from significant ML error (61.77±24.39%). In the Sup. Post. Ipsil. octant, both cameras exhibited lower agreement (frontal: 46.58±35.17%; offset: 51.14±27.03%) with high AP directional errors (frontal: 40.11±34.28%; offset: 34.96±24.53%). For the Inf. Ant. Ipsil. octant, both configurations achieved high agreement (frontal: 94.32±3.44%; offset: 95.21±2.21%) with consistently low directional errors. In the Inf. Ant. Contra. octant, the frontal camera demonstrated higher agreement (92.79±5.62%) than the offset camera (46.86±30.49%), though the offset camera showed higher ML error (38.65±29.24%). Finally, in the Inf. Post. Ipsil. octant, both cameras demonstrated moderate agreement (frontal: 67.11±14.43%; offset: 64.89±23.45%) with elevated AP directional errors (frontal: 27.16±15.60%; offset: 31.48±25.81%).

No statistically significant differences in the percentage workspace reached were observed between the frontal-configuration MMC reconstructions and the marker-based reference. The offset camera configuration, however, differed significantly from the marker-based system and from the frontal configuration in the contralateral anterior octants (Sup. Ant. Contra. and Inf. Ant. Contra.; Table 2).

Agreement analysis (Figure 3) showed high agreement (>90%) between the frontal camera and the marker-based system in four of six octants, primarily in anterior regions. Reduced agreement and larger directional errors were observed in posterior-facing octants for both systems, predominantly in the AP direction.

## 4. Discussion

This study demonstrates that a monocular MMC approach can accurately quantify the percentage workspace reached during the UERW task in healthy adults, and provides a technical foundation for further work in clinical populations. The UERW measurements obtained using the frontal-camera system were comparable to those derived from a traditional marker-based motion capture system, supporting the feasibility of monocular MMC for multi-planar, clinically relevant assessments. To our knowledge, this is the first demonstration that a monocular system can achieve performance similar to a marker-based reference for clinical evaluation of upper extremity mobility, whereas previous work has primarily validated the underlying measurement accuracy of multi-camera systems rather than clinical outcome measures [17,22]. Other monocular approaches, such as [30,31,32], are limited by a lack of anatomically accurate model for clinical decision making, but have been, nonetheless, shown to be valid tools for the assessment of UE tasks with monocular cameras. By reducing technical complexity, equipment requirements, and setup time, monocular MMC offers a practical solution for implementing quantitative UE mobility assessments in small clinical teams or point-of-care settings, potentially increasing accessibility and adoption of objective movement analysis in routine practice.

An important consideration in this analysis is the definition of peak reach distance, which was computed post hoc from both the MMC and marker-based reconstructions. This parameter determines the virtual target locations for each participant for the post hoc analysis and differs slightly from the VR target placement that is seen by the participants in the headset. We observed small differences between systems in peak reach distance (0.04±0.03 m for both orientations), which are likely attributable to systematic biases in keypoint localization. Because the detected keypoints are not rigidly attached to anatomical landmarks, small shifts in detection can propagate through the reconstruction pipeline. Nevertheless, given the minimal differences in peak reach distance, subsequent differences in UERW were primarily driven by camera orientation.

Errors in both systems were driven largely by the inherent difficulty of measuring motions that were out of line with the view of the monocular video. The frontal camera demonstrated its poorest agreement in the two posterior octants, where depth ambiguity produced elevated AP-direction errors (Figure 3). The offset camera showed a comparable trend, with substantial AP-direction errors in posterior octants and ML-direction errors in the two contralateral octants. These patterns corroborate previous research showing that measurements are most accurate in the anatomical plane that aligns with the camera view [27]. The concentration of error in the posterior and contralateral octants suggests a sensitivity to perspective projection constraints. Specifically, when movement vectors align with the camera’s optical axis, the resulting foreshortening reduces the signal-to-noise ratio of 2D keypoint displacement. This creates a reliance on the biomechanical model’s depth estimation, which, as evidenced by our AP-direction error rates, struggled to resolve depth ambiguity in the absence of binocular or multi-view parity.

### 4.1. Frontal Camera

Overall, however, the frontal camera orientation showed no statistically significant difference from the marker-based assessment and may serve as a viable clinical solution for the assessment of UERW, particularly for the anterior workspace. Deviations were typically less than five targets per octant, well below deficits observed in upper extremity clinical populations, where differences between affected and unaffected limbs have been shown to be up to 35% of the workspace per octant reached in pediatric patients with brachial plexus palsy. The only exceptions were the Inf. Ant. Ipsil. and Contra. octants, which had differences of less than 10% of the workspace per octant [2]. With our cohort, we are well below the differences observed in clinical populations. For reaches into the superior anterior octants, which require shoulder elevation, a function commonly impaired in patient groups such as stroke survivors and children with brachial plexus palsy, the frontal camera configuration performed well, showing no significant difference from marker-based motion capture in any of the four anterior octants [8,43,44]. This is consistent with prior findings for tasks conducted within the same workspace [17,22]. The observed mean bias of less than 5% for the frontal configuration is well within the acceptable clinical range, as it is significantly smaller than the 20–50% deficits typically used to differentiate between patient populations and healthy controls [2,10].

### 4.2. Offset Camera

In contrast, the offset camera orientation significantly underestimated the number of percentage workspace reached in several key regions, including the superior–anterior contralateral, inferior–anterior contralateral, and inferior–posterior ipsilateral octants (Figure 2). These discrepancies underscore a fundamental trade-off in monocular motion capture between perspective-dependent depth resolution and anatomical occlusion. While our findings support the general feasibility of monocular systems, they highlight that no single viewpoint is currently optimal for tasks requiring dynamic, multi-planar motion that aligns with the camera’s optical axis. For example, the offset camera achieved less than 40% agreement in the superior–anterior contralateral octant, exhibiting large ML errors. Mechanistically, this poor performance is triggered by cross-body movements where the torso or the reaching arm itself physically obstructs the camera’s line of sight to the hand and wrist. During these periods of occlusion, the monocular reconstruction must “infer” distal joint positions, leading to inaccurate depth estimations. This stands in stark contrast to the Inf. Ant. Ipsil. octant, where both camera configurations achieved high agreement rates (>95%). In this zone, the movement occurs largely within the camera’s optimal plane of view, minimizing both occlusion and projection foreshortening. Consequently, the frontal camera showed minimal bias (1.22% mean difference), whereas the offset camera’s persistent underestimation in other zones reflects the current algorithmic difficulty in resolving depth when the limb’s movement vector parallels the camera lens.

While this study demonstrates the technical feasibility of monocular MMC, several constraints must be addressed to ensure clinical utility. First, the generalization of these findings is limited by the homogeneity of the study cohort, which consisted entirely of healthy adults. Clinical populations, such as stroke survivors or children with brachial plexus palsy, often exhibit atypical movement kinematics, including tremors, slower velocities, or compensatory trunk movements. It remains to be determined if the current detection algorithms maintain their level of precision when faced with these neuromotor pathologies, as the “negligible” errors observed here—in fewer than 5% of the workspace per octant—may have greater functional implications in patients. For instance, in severe brachial plexus injuries where a patient may only reach the inner margins of the workspace, a small depth estimation error could be the deciding factor in whether a target is classified as “reached”.

### 4.3. Limitations

While the monocular approach significantly reduces the equipment requirements and setup time associated with traditional marker-based systems and can be recorded from a single mobile device, several limitations remain. Future clinical implementation studies must evaluate the effects of anatomical deformities, varying body sizes, and lighting conditions to define the operational limits of monocular MMC for UERW assessment, as this study was only a technical validation on nondisabled adults.

The analysis was conducted post hoc, which limits immediate utility at the point of care; for monocular MMC to be successfully integrated into routine clinical workflows, the transition from data capture to actionable reporting must be streamlined. Furthermore, the high regional variability observed—with average percent errors reaching as high as 15.45% for the frontal camera and 50.21% for the offset view in contralateral octants—suggests that while the system is suitable for assessing total reachable workspace, it may lack the consistency required for precise localized analysis in superior or contralateral octants. Future iterations should focus on integrating real-time feedback and leveraging mobile platforms to provide immediate biofeedback during assessment, thereby reducing technical overhead and increasing accessibility in both busy clinics and home-based rehabilitation settings. Joint kinematics were not assessed in this study, as they are not standard metrics for the UERW task. Addressing these spatial and algorithmic limitations remains a prerequisite for expanding the reach of objective movement analysis in diverse clinical environments.

### 4.4. Conclusions

This study provides the first technical feasibility assessment of a monocular, video-based system for analyzing the UERW task. Our results in a cohort of nine adults without movement impairments indicate that a frontal camera orientation can yield UERW measurements comparable to gold-standard marker-based motion capture, particularly within the anterior octants. While these findings demonstrate the technical validity of a monocular camera setup as a potential alternative to complex, multi-camera systems, this study serves as a preliminary proof of concept rather than a broad clinical validation.

## Figures and Tables

**Table 1:** Comparison of the total percentage workspace reached in each octant for each motion capture system. Values are reported as mean ± standard deviation (SD). Higher values indicate greater workspace accessibility. This table provides the primary clinical outcome used for the ANOVA analysis.

Octant	System	Mean ± SD (%)
Sup. Ant. Ipsil.	Marker-Based	86.44 ± 8.63
	Frontal	81.67 ± 9.27
	Offset	78.11 ± 11.73
Sup. Ant. Contra.	Marker-Based	25.89 ± 11.52
	Frontal	29.89 ± 9.55
	Offset	12.89 ± 8.04
Sup. Post. Ipsil.	Marker-Based	11.78 ± 8.15
	Frontal	10.11 ± 9.68
	Offset	9.89 ± 10.36
Inf. Ant. Ipsil.	Marker-Based	82.78 ± 6.91
	Frontal	84.00 ± 5.74
	Offset	80.11 ± 7.24
Inf. Ant. Contra.	Marker-Based	15.22 ± 9.77
	Frontal	17.44 ± 6.82
	Offset	9.89 ± 6.51
Inf. Post. Ipsil.	Marker-Based	23.11 ± 11.06
	Frontal	23.89 ± 13.44
	Offset	18.89 ± 10.75

**Table 2:** Pairwise comparisons of percentage of reachable workspace between configurations. Values represent *p*-values and partial eta squared effect sizes ($\eta_{p}^{2}$). Statistically significant results (p<0.05, Bonferroni-corrected) indicate differences in workspace estimation between systems.

Octant	Offset/Marker	Frontal/Marker	Offset/Frontal
	p/$\eta_{p}^{2}$	p/$\eta_{p}^{2}$	p/$\eta_{p}^{2}$
Inf. Ant. Contra.	0.063 / 0.507	0.689 / 0.175	0.000 / 0.892
Inf. Ant. Ipsil.	0.522 / 0.218	0.999 / 0.070	0.215 / 0.350
Inf. Post. Ipsil.	0.075 / 0.486	0.999 / 0.068	0.056 / 0.520
Sup. Ant. Contra.	0.000 / 0.854	0.320 / 0.292	0.000 / 0.886
Sup. Ant. Ipsil.	0.236 / 0.337	0.303 / 0.300	0.406 / 0.256
Sup. Post. Ipsil.	0.757 / 0.160	0.551 / 0.209	0.999 / 0.002

**Table 3:** Bland–Altman statistics comparing percentage of reachable workspace between marker-based motion capture and two MMC configurations. Mean difference is calculated as (MMC − marker-based); negative values indicate underestimation by MMC.

Octant	Camera	Mean Diff. (%)	95% CI (%)
All Octants	Frontal	−0.70	−11.70 to 12.90
	Offset	−5.59	−19.90 to 8.71
Sup. Ant. Ipsil.	Frontal	−4.78	−18.87 to 9.31
	Offset	−8.33	−31.57 to 14.90
Sup. Ant. Contra.	Frontal	4.00	−11.25 to 19.25
	Offset	−13.00	−23.28 to −2.72
Sup. Post. Ipsil.	Frontal	−1.67	−7.86 to 4.53
	Offset	−1.89	−10.51 to 6.74
Inf. Ant. Ipsil.	Frontal	1.22	−8.06 to 10.51
	Offset	−2.67	−13.18 to 7.84
Inf. Ant. Contra.	Frontal	2.22	−9.03 to 13.47
	Offset	−5.33	−16.07 to 5.40
Inf. Post. Ipsil.	Frontal	0.78	−10.60 to 12.15
	Offset	−4.22	−12.92 to 4.48

## Data Availability

The data presented in this study are not publicly available due to privacy restrictions associated with the collection of human movement data at a pediatric clinical institution. Data may be made available from the corresponding author upon reasonable request.

## Abbreviations

The following abbreviations are used in this manuscript:

UERW	Upper Extremity Reachable Workspace
AI	Artificial intelligence
MMC	Markerless Motion Capture
PROs	Patient-reported outcomes
UE	Upper extremity
AP	Anterior posterior
ML	Medial–lateral;
SI	Superior–inferior
Sup. Ant. Ipsil.	Superior anterior ipsilateral
Sup. Ant. Contra.	Superior anterior contralateral
Inf. Ant. Ipsil.	Inferior anterior ipsilateral
Inf. Ant. Contra.	Inferior anterior contralateral
Inf. Post. Ipsil.	Inferior posterior ipsilateral
Sup. Post. Ipsil.	Superior posterior ipsilateral
ANOVA	Analysis of variance
CIs	Confidence intervals
